# A novel population balance model for the dilute acid hydrolysis of hemicellulose

**DOI:** 10.1186/s13068-015-0211-5

**Published:** 2015-02-19

**Authors:** Ava A Greenwood, Troy W Farrell, Zhanying Zhang, Ian M O’Hara

**Affiliations:** Mathematical Sciences, Queensland University of Technology, 2 George Street, Brisbane, 4001 QLD Australia; Centre for Tropical Crops and Biocommodities, Queensland University of Technology, 2 George Street, Brisbane, 4001 QLD Australia

**Keywords:** Pretreatment, Hemicellulose, Acid hydrolysis, Sugarcane bagasse, Mathematical modelling, Kinetics, Hard-to-hydrolyse

## Abstract

**Background:**

Acid hydrolysis is a popular pretreatment for removing hemicellulose from lignocelluloses in order to produce a digestible substrate for enzymatic saccharification. In this work, a novel model for the dilute acid hydrolysis of hemicellulose within sugarcane bagasse is presented and calibrated against experimental oligomer profiles. The efficacy of mathematical models as hydrolysis yield predictors and as vehicles for investigating the mechanisms of acid hydrolysis is also examined.

**Results:**

Experimental xylose, oligomer (degree of polymerisation 2 to 6) and furfural yield profiles were obtained for bagasse under dilute acid hydrolysis conditions at temperatures ranging from 110°C to 170°C. Population balance kinetics, diffusion and porosity evolution were incorporated into a mathematical model of the acid hydrolysis of sugarcane bagasse. This model was able to produce a good fit to experimental xylose yield data with only three unknown kinetic parameters *k*_*a*_,*k*_*b*_ and *k*_*d*_. However, fitting this same model to an expanded data set of oligomeric and furfural yield profiles did not successfully reproduce the experimental results. It was found that a “hard-to-hydrolyse” parameter, *α*, was required in the model to ensure reproducibility of the experimental oligomer profiles at 110°C, 125°C and 140°C. The parameters obtained through the fitting exercises at lower temperatures were able to be used to predict the oligomer profiles at 155°C and 170°C with promising results.

**Conclusions:**

The interpretation of kinetic parameters obtained by fitting a model to only a single set of data may be ambiguous. Although these parameters may correctly reproduce the data, they may not be indicative of the actual rate parameters, unless some care has been taken to ensure that the model describes the true mechanisms of acid hydrolysis. It is possible to challenge the robustness of the model by expanding the experimental data set and hence limiting the parameter space for the fitting parameters. The novel combination of “hard-to-hydrolyse” and population balance dynamics in the model presented here appears to stand up to such rigorous fitting constraints.

## Background

Sugarcane bagasse is a promising feedstock for the production of second-generation bioethanol, whereby the cellulosic material within bagasse is hydrolysed by enzymes to produce glucose, which is subsequently fermented to produce bioethanol [1,2]. Bagasse fibres are structurally complex, comprised of three key materials: cellulose, hemicellulose and lignin. Hemicellulose forms a monolayer coating around cellulose and thus inhibits the enzymatic saccharification process [3,4]. Acid pretreatment is a method of removing hemicellulose from bagasse by hydrolysing the linkages between the monomeric units of the hemicellulose polymers. This allows enzymes greater access to the cellulosic material. Ensuring the efficiency of acid pretreatment improves the overall cost-effectiveness of bioethanol production from second-generation feedstocks [5].

Mathematical models can prove to be useful in testing the impact of varying reaction conditions upon a chemical system, with a significant time and cost saving compared to experimentation. Models may also help to inform the influence that certain input parameters and experimental conditions have upon the reaction outcomes. However, the ability of a model to both represent the chemical and physical behaviours of a system and to predict experimental outcomes must be carefully scrutinised. Without testing the robustness of a model, there may be little confidence attached to its outcomes.

A simple model of acid hydrolysis is the Saeman kinetic model in which hemicellulose is hydrolysed to form xylose, which in turn produces its own degradation products [6]. A particularly enduring variation of this model was derived by Kobayashi and Sakai in which the bagasse is portioned into two subsets, one fast hydrolysing and the other slow to hydrolyse [7,8]. Hereafter, this model is referred to as the “hard-to-hydrolyse” model. Often an oligomeric phase is introduced into these models, or a full oligomeric spectrum may be obtained through the population balance framework of Simha [9]. Such models allow for the inclusion of chain-dependent phenomena in the model, such as solubility and diffusivity. The authors have previously incorporated diffusivity and time-dependent porosity calculations into a population balance model of microscale acid hydrolysis [10]. This model was used to propose constraints on model fitting parameters but was not predictive due to the small size scale, which limited the scope for experimental validation.

In this work, we propose a fibre scale model that marries the chain length dependency of population balance equations with “hard-to-hydrolyse” kinetics. This new model also accounts for the diffusion of species from within the fibre into the surrounding hydrolysate and allows for the porosity of the material to vary temporally and spatially. The model parameters were determined by simultaneously fitting the model to experimentally obtained xylose and oligomer yield profiles (degree of polymerization (DP) 2 to 6) as well as the yield of furfural, a degradation product of xylose. By comparing the model results to oligomer profiles in addition to the monomer (xylose) yield curve, the variability associated with the model parameters is restricted. Hence, it is hypothesised that the resultant parameters carry more weight than those which have not been subjected to an equivalently stringent fitting process.

### Model development

**Figure 1:**
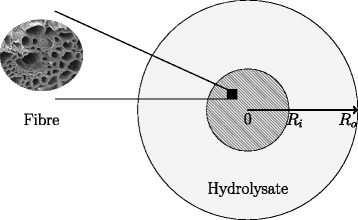
**Schematic of the fibre scale domain.** The domain consists of two regions, the fibre (0 ≤*r*≤*R*_*i*_) and the hydrolysate (*R*_*i*_<*r*≤*R*_*o*_). Untreated bagasse SEM image (×600 magnification) courtesy of Dr. Thor Bostrom *Electron Microscopy Facility* and Dr. John Knight *Mathematical Sciences*, Queensland University of Technology (2008).

Experimentally, acid hydrolysis was conducted by loading 5 g of dried sugarcane bagasse into a 66-mL Dionium™ cell, which was subsequently filled with 0.5 wt *%* sulfuric acid. To define the model domain, it is assumed that the hydrolysis of each individual bagasse fibre in the cell is independent of any other. Hence, the simplifying assumption is made that the hydrolysis of all the materials in the cell can be approximated by a model of the hydrolysis of a single bagasse fibre. Thus, consider a single idealised, cylindrical bagasse fibre surrounded by an associated volume of hydrolysate. Since the length of a bagasse fibre is considerably longer than its radius, it is assumed that transport in the longitudinal direction is comparatively slow. Consequently, only the radial cross section of the fibre and the associated region of hydrolysate is considered, as per the schematic in Figure 1. We assume reaction and one-dimensional transport across two distinct regions of the domain, the fibre (0≤*r*≤*R*_*i*_) and the hydrolysate (*R*_*i*_<*r*≤*R*_*o*_). The fibre component of the domain is represented by an initially homogenous distribution of lignocellulosic biomass and pores filled with an acid solution (H_3_O^+^). Within the fibre, the volume fraction of the solid biomass is denoted by *ε*_*s*_ and the volume fraction of the acid filled pores is labelled *ε*_*v*_. The hydrolysate contains only the acid solution and hence in this region of the domain *ε*_*v*_=1 throughout.

**Figure 2:**
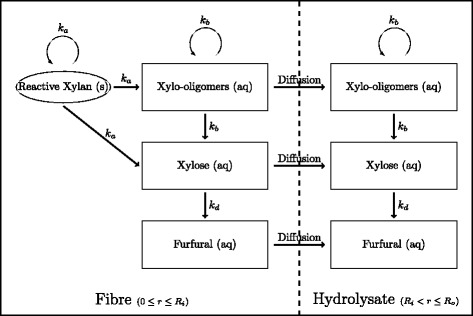
**Diagram of the kinetic and mass transport mechanisms present in the model.** Reaction pathway depicting scission of hemicellulose in the fibre and hydrolysate and diffusion of chains from the fibre into the hydrolysate.

Figure 2 demonstrates the reactive and transport processes included in the model, namely the acid hydrolysis and diffusion of hemicellulose chains both within the fibre and in the hydrolysate. Initially, all hemicelluloses are contained in the fibre as xylan (solid chains of length *m*<*i*≤*N*). However, as scission proceeds, soluble chains of length *m* or less are produced and hence able to diffuse through the fibrous material and into the surrounding region of hydrolysate, where they may continue to be hydrolysed by the acid solution. The rate constants *k*_*a*_ and *k*_*b*_ (m^3^mol^−1^s^−1^) represent the rate of hydrolysis of solid chains (*i*=*m*+1,…,*N*) and aqueous chains (*i*=2,…,*m*), respectively. The rate of furfural production caused by xylose degradation is given by *k*_*d*_ (m ^3^ mol ^−1^ s ^−1^). Consequently, only aqueous hemicellulose oligomers (chains of length *i*=1,…,*m*) and their degradation products may be present in the hydrolysate. Therefore, there are a different number of species modelled in each region of the domain as demonstrated in Figure 2. The model also accounts for the change in the porosity of the fibrous material caused by the solubilisation of solid xylan chains.

A discrete population balance approach is used to account for the chain degradation kinetics by formulating chain scission as a series of polymer degradation equations [9]. This methodology enables hemicellulose chains of all lengths to be explicitly counted, which allows for the inclusion of chain-length dependent solubility and diffusion [10]. Time-dependent polydispersity information was also collected due to the population balance equations, which provided a more stringent set of criteria to be used when parameter fitting the rate constants.

In the literature, the existence of a fast and slow hydrolysing hemicellulose fraction is readily observed [7]; however, the population balance framework does not readily allow for Kobayashi and Sakai’s separate “hard-to-hydrolyse” and “easy-to-hydrolyse” classes to be incorporated into the model [8]. In order to best approximate this phenomenon while maintaining full chain length dependence, it is assumed that the rate of hydrolysis of the slow component of hemicellulose is effectively zero on the timescale of the fast hydrolysis reaction. Consequently, there exists an unreactive portion of bagasse, (*α*(*T*)), and a hydrolysable portion, (1−*α*(*T*)), as an alternative to the easy and hard-to-hydrolyse kinetic model, where *T* represents temperature (K). A similar parameter has been used in conjunction with the Saeman kinetic model by Bustos *et al.* [11], and Zhao *et al.* have developed a parameter to represent the “potential hydrolysis degree” which is alike in interpretation to (1−*α*) [12]. Yan *et al.* also use a similar ratio to describe an unreactive component of cellulose in a model of cellulosic acid hydrolysis; however, the interpretation of this parameter is not the same in this context [13].

The fibre model was adopted from the author’s previous cell wall acid hydrolysis model [10]. In this work, cylindrical coordinates were utilised (rather than cartesian coordinates) to better represent the radial cross section of the fibre. The fibre equation system for the region (0≤*r*≤*R*_*i*_) is therefore:

(1) $$ {\fontsize{8.5}{12}\begin{aligned} \frac{\partial\phi_{F}}{\partial{t}}= \underbrace{k_{d}\psi_{H^{+}}\phi_{1}}_{\text{Formation}} + \underbrace{\frac{1}{r}\frac{\partial}{\partial{r}} \left(r D^{F}_{\text{eff}}(\epsilon_{v}) \frac{\partial\phi_{F}}{\partial{r}} \right)}_{\text{Diffusion}}, \end{aligned}}  $$

(2) $$ {\fontsize{8.5}{12}\begin{aligned} \frac{\partial\phi_{1}}{\partial{t}}&=\underbrace{- k_{d}\psi_{H^{+}}\phi_{1}}_{\text{Degradation}} + \underbrace{2k_{a}\psi_{H^{+}}\sum_{j = m+1}^{N}\Omega_{1,j-1}\phi_{j}}_{\text{Formation from solid chains}}\\ &\quad+ \underbrace{2k_{b}\psi_{H^{+}}\sum_{j = 2}^{m}\Omega_{1,j-1}\phi_{j}}_{\text{Formation from aqueous chains}} +\, \underbrace{\frac{1}{r}\frac{\partial}{\partial{r}}\left(r D_{\text{eff}}(\epsilon_{v}) \frac{\partial{\phi_{1}}}{\partial{r}} \right)}_{\text{Diffusion}}, \end{aligned}}  $$

(3) $$ {\fontsize{8.5}{12}\begin{aligned} \frac{\partial{\phi_{i}}}{\partial{t}} &= \underbrace{-k_{b}\psi_{H^{+}}\phi_{i}}_{\text{Scission}} \;+ \underbrace{2k_{a}\psi_{H^{+}}\sum\limits_{j = m+1}^{N}\Omega_{i,j-i}\phi_{j}}_{\text{Formation from solid chains}}\\ &\quad+ \underbrace{2k_{b}\psi_{H^{+}}\sum\limits_{j = i+1}^{m}\Omega_{i,j-i}\phi_{j}}_{\text{Formation from aqueous chains}}\\ &\quad+ \, \underbrace{\frac{1}{r}\frac{\partial}{\partial{r}}\left(r D_{\text{eff}}(\epsilon_{v}) \frac{\partial{\phi_{i}}}{\partial{r}} \right)}_{\text{Diffusion}}, \quad (i = 2,3,\ldots,m-1, m) \end{aligned}}  $$

(4) $$ {\fontsize{8.5}{12}\begin{aligned} \frac{\partial{\phi_{i}}}{\partial{t}} &= \underbrace{-k_{a}\psi_{H^{+}}\phi_{i}}_{\text{scission}}\\ &\quad+ \, \underbrace{2k_{a}\psi_{H^{+}}\sum\limits_{j = i+1}^{N}\Omega_{i,j-i}\phi_{j}}_{\text{Formation from solid chains}}, \, (i = m+1,m+2,\ldots,N-1, N) \end{aligned}}  $$

and

(5) $$ {\fontsize{8.5}{12}\begin{aligned} \epsilon_{v} = 1 - \left(\hat{F} + \sum\limits_{i = m+1}^{N} \epsilon_{i} +\epsilon_{\alpha} \right). \end{aligned}}  $$

where Equations 1 through 4 describe the time (*t*) rate of change of the volume averaged concentrations of furfural, *ϕ*_*F*_ (kg m ^−3^), xylose, *ϕ*_1_ (kg m ^−3^), aqueous oligomers, *ϕ*_*i*_*i*=2,…,*m* (kg m ^−3^) and reactive xylan, *ϕ*_*i*_*i*=*m*+1,…,*N* (kg m ^−3^), respectively. Here, $\phantom {\dot {i}\!}\psi _{H^{+}}$ (mol m ^−3^) is the effective acid concentration given by $\epsilon _{v} C_{H^{+}}\phantom {\dot {i}\!}$. It is assumed that only one mole of hydrogen ions is liberated from one mole of sulfuric acid [14,15]. The reaction rate constants *k*_*a*_, *k*_*b*_ and *k*_*d*_ are demonstrated in Figure 2. The parameter *Ω*_*i*,*j*−*i*_ is the breakage kernel (from the population balance kinetics), and *D*_eff_(*ε*_*v*_) (m ^2^*s*^−1^) represents an effective diffusion coefficient used to account for the tortuous nature of the bagasse fibre interior. Xylan was taken to have a maximum chain length of *N*=100. Although this falls within the range of the expected degree of polymerisation of hemicellulose (DP 80-200), the exact choice of *N*=100 was motivated by convenience [3].

Arrhenius kinetics of the form:

(6) $$ k_{a,b,d} = k_{a,b,d}^{0} \, \text{exp}\left(\frac{-E_{a_{a,b,d}}}{RT} \right)  $$

were used to describe the rates of reaction. A modified Stokes-Einstein approximation to the diffusion coefficient was used for the effective diffusion coefficient, *D*_eff_(*ε*_*v*_) (m ^2^*s*^−1^), such that:

(7) $$ D_{\text{eff}}(i,\epsilon_{v}) = {\epsilon_{v}^{3}} D_{\infty}(i), \qquad D_{\infty}(i) = \frac{k_{B}T}{6\pi\eta R_{h}(i)},  $$

where ${\epsilon _{v}^{3}}$ accounts for the tortuous nature of the fibre [16,17], *k*_*B*_ (m^2^ kg s^−2^ K^−1^) is Boltzmann’s constant, *η* (kg m ^−1^ s ^−1^) is the dynamic viscosity of the acid solution and $R_{h}(i) = 0.676l\sqrt {i} $ (m) is the hydrodynamic radius of polymer chains of length *i* in solution. A detailed description of the derivation of the model and these auxillary equations can be found in [10].

Equation 5 describes the porosity of the fibre as it evolves over time. It was assumed that the porosity of the fibre was initially 25.4 *%* (*v*/*v*) based on the porosity measurement of rice hulls [18]. Although the porosity of sugarcane bagasse has been reported in the literature, a measure of porosity as a volume fraction is specifically required for this model due to the volume averaged nature of the equations [19,20]. Sugarcane bagasse and rice hulls are both lignocellulosic agricultural residues, and hence, it is assumed that the porosity of rice hulls provides a reasonable substitute. It is difficult to validate or reject this assumption based on the SEM image of bagasse in Figure 1, since the orientation of the image and cell type featured may distort the apparent porosity [21]. The parameters $\hat {F}$ and *ε*_*α*_ represent the fixed volume fractions of lignocellulose and unreactive hemicellulose, respectively. The unreactive portion of hemicellulose is defined such that if the initial total volume fraction of hemicellulose is ${\epsilon _{N}^{0}}$ (assuming an initially monodisperse state, for simplicity), then $\epsilon _{\alpha } = \alpha (T){\epsilon _{N}^{0}}$. The initial volume fractions of lignin, cellulose and xylan make up the remaining non-porous 74.6 *%* of the bagasse material. The initial volume fractions of cellulose, lignin and xylan were determined so as to preserve the ratio of components determined experimentally. Although these experiments measured the mass fraction of each component, the composition values were assumed to be a suitable substitute for volume fractions since the densities of lignin and hemicellulose cannot be determined.

The hydrolysate model is stated similarly to the fibre model with two notable exceptions. Firstly, as indicated in Figure 2, all insoluble chains of length *i*=*m*+1,…,*N* are omitted. Secondly, the void volume fraction is equivalent to the total volume of the region, *ε*_*v*_=1, and hence is not explicitly stated in the equations.

The hydrolysate model equations for the region (*R*_*i*_<*r*≤*R*_*o*_) are therefore:

(8) $$ {\fontsize{8.9}{12}\begin{aligned} \frac{\partial\phi_{F}}{\partial{t}} = \underbrace{k_{d}\psi_{H^{+}}\phi_{1}}_{\text{Formation}} + \underbrace{\frac{1}{r}\frac{\partial}{\partial{r}}\left(r D^{F}_{\infty} \frac{\partial\phi_{F}}{\partial{r}} \right)}_{\text{Diffusion}}, \end{aligned}}  $$

(9) $$ {\fontsize{8.9}{12}\begin{aligned} \frac{\partial\phi_{1}}{\partial{t}} &= \underbrace{- k_{d}\psi_{H^{+}}\phi_{1}}_{\text{Degradation}} + \,\underbrace{2k_{b}\psi_{H^{+}}\sum_{j = 2}^{m}\Omega_{1,j-1}\phi_{j}}_{\text{Formation from aqueous chains}}\\ &+\, \underbrace{\frac{1}{r}\frac{\partial}{\partial{r}}\left(r D_{\infty}(i) \frac{\partial\phi_{1}}{\partial{r}} \right)}_{\text{Diffusion}}, \end{aligned}}  $$

(10) $$ {\fontsize{8.9}{12}\begin{aligned} \frac{\partial\phi_{i}}{\partial{t}} &= \underbrace{-k_{b}\psi_{H^{+}}\phi_{i}}_{\text{Scission}} \,+\, \underbrace{2k_{b}\psi_{H^{+}}\sum_{j = i+1}^{m}\Omega_{i,j-i}\phi_{j}}_{\text{Formation from aqueous chains}}\\ &\quad+ \, \underbrace{\frac{1}{r}\frac{\partial}{\partial{r}}\left(r D_{\infty}(i) \frac{\partial\phi_{i}}{\partial{r}} \right)}_{\text{Diffusion}}, (i = 2,3,\ldots,m-1, m). \end{aligned}}  $$

In this work, we take the radial distance of the hydrolysate, *R*_*o*_−*R*_*i*_ to be 2.32 times that of the fibre length, *R*_*i*_. This was determined experimentally, whereby the volume of the hydrolysate pumped into the reactor was on average ten times the total volume of the bagasse fibres.

At the centre of the fibre, *r*=0, a no-flux boundary condition is required due to the symmetry of the domain about this point. At the outer boundary (the outer edge of the hydrolysate), *r*=*R*_*o*_, a no-flux condition is again imposed, in order to represent the closed system of the reaction vessel (all mass is expected to be contained within the fibre and the surrounding hydrolysate). Consequently, the boundary conditions become:

(11) $$ \frac{\partial\phi_{i}}{\partial{r}}(0,t) \quad\!\! = \!\!\quad \frac{\partial\phi_{i}}{\partial{r}} (R_{o},t) \quad =\quad 0, \qquad (i = 1,2,\ldots m).  $$

Assuming an initially monodisperse distribution of chains of length *N*, the initial condition for reactive hemicellulose is given by:

(12) $$ \phi_{i} (r,0) =\begin{cases} (1-\alpha(T)){\epsilon_{N}^{0}}\rho_{s}\,, & \text{if}\quad i = N\\ 0\,, & \text{if}\quad i < N. \end{cases}  $$

In addition to those already outlined, there are five further assumptions implicit in the model equations. Firstly, it is assumed that xylan is a suitable representative for hemicellulose since xylose is typically the primary constituent of hemicellulose in bagasse [22]. Furthermore, the xylan is considered to exist as a linear chain of xylose monomers without any side chains or branches. This simplification was necessitated by the use of population balances which require the probability of scission at each chain linkage to be known. An equal probability of scission at all sites can be assumed under the simplified conditions. It is difficult to quantify the scission probabilities when non-homogeneities are introduced by the geometry of the polymer and the distribution of different functional groups across the chain. Furthermore, it is assumed that the degradation products of xylose are best represented by furfural and that the further degradation of furfural can be ignored. In support of this assumption, neither of the two prominent acid-catalysed degradation products of furfural, formic acid or levulinic acid, was recorded experimentally during analysis of the hydrolysate [23]. Since hemicellulose is approximated by linear xylan, there is no consideration for acetyl groups in the model either [24]. This imposes the third assumption that the acetic acid liberated from acetyl groups in the hemicellulose does not affect the acid concentration catalysing the hydrolysis reaction and hence can be excluded from the model equations. This assumption is further rationalised by comparing the p*K*a (at standard lab conditions) of acetic acid, 4.76, and the hydronium ion, 0.0 [25]. Given that acetic acid is the weaker acid, it suggests that equilibrium does not lie in favour of strong dissociation of the acetic acid. It is further assumed that the fraction of the total volume occupied by cellulose and lignin is unchanged under the conditions of dilute acid hydrolysis. This assumption is necessary in order to formulate the porosity equation. While it would be of merit to incorporate the reaction kinetics of all the three key constituents of bagasse into the model, this is again complicated by the population balances since the scission of such a complex network of interlinking chains is difficult to conceptualise in terms of scission probabilities and solubility. Consequently, it remains beyond the scope of this work to model cellulose and lignin degradation. Finally, it is assumed that the domain is isothermal and isobaric. The parameters used to produce the model results are displayed in Table 1.

**Table 1:** **Parameter values used for model simulation**

**Parameter**	**Value**	**Units**	**Ref**
$C_{H^{+}}\phantom {\dot {i}\!}$	51	mol m^−3^	-
	0.5	wt *%*	
*R*	8.314	J K ^−1^ mol ^−1^	[26]
*R*_*i*_	3.75×10^−4^	m	-
$D^{F}_{\infty }$	1.12×10^−9^	m ^2^ s ^−1^	[27]
$\hat {F}$	0.581	-	-
*N*	100	-	[3]
*m*	15	-	[28]
*k*_*B*_	1.38×10^−23^	m^2^ kg s^−2^ K^−1^	[26]
*l*	0.65×10^−9^	m	[29]
${\epsilon _{N}^{0}}$	0.165	-	-

The model yield was calculated by summing across all of the spatial points in the relevant regions of the domain such that:

(13) $$ Y_{i}(t) = \frac{\sum_{k=\hat{R}_{i}}^{\hat{R}_{o}} r_{k} \phi_{i,k}(t) \Delta r_{k}}{\sum_{k=1}^{\hat{R}_{i}} r_{k} (\phi_{N\!,\,k}(0)+ \epsilon_{\alpha} \rho_{s})\Delta r_{k} },  $$

where $\hat {R}_{i}$ and $\hat {R}_{o}$ represent the number of spatial nodes in the fibre and total domain, respectively. Water was assumed to be in excess and was not modelled explicitly. Consequently, in the model, the mass of a xylose monomer does not increase by the weight of a water molecule when scised. In reality, the yield of xylose as a mass fraction could be greater than 100*%* due to the addition of the water, and hence, the experimental yield must be corrected as described in the Methods section. The efficacy of this model was determined by fitting the model yields, *Y*_*i*_, to the experimentally obtained oligomer profiles.

## Results and discussion

Dilute acid pretreatments were conducted with 0.5% H_2_SO_4_ at five different temperatures ranging between 110°C and 170°C. For each temperature, a time series of yields was obtained for furfural, xylose and oligomers from xylobiose to xylohexaose (*X*_2_ – *X*_6_). The maximum xylose yields obtained for each temperature ranged from 63.2 *%* at 110°C after 360 min to 92.1 *%* obtained at 155°C after 20 min. The maximum oligomer and furfural yields recorded were significantly smaller than those obtained for xylose, although some appreciable amounts of the shorter oligomer chains were recorded.

These experimental results were used to first calibrate and then validate the model presented in the Model Development section. The calibration was necessary in order to identify suitable values for the unknown model parameters by fitting the model to the experimental data collected at 110°C, 125°C and 140°C, respectively. The hemicellulose yields recorded at 155°C and 170°C were not used to fit rate parameters and hence do not appear in the Arrhenius plot discussed below. This is because the experimental yields measured at 155°C and 170°C were compromised by the automated heating time of the Dionex™ ASE™ 350. The heat up time of the solvent extractor was long compared to the timescale of acid hydrolysis at these higher temperatures; hence, the yields measured at zero static time (that is, at *t*=0) were non-zero to a statistically significant degree. Calculations exist in the literature to distinguish the yield due to the preheating time from the true experimental yield; however, these calculations are typically based on simpler kinetics [30]. An investigation into such calculations for non-linear population balance kinetics may provide an interesting future course of enquiry.

The calibration was completed using PEST, a model-independent parameter estimation tool. PEST uses the Gauss-Marquardt-Levenberg method to find values of the fitting parameters that minimise the discrepancies between the model results and the experimental data via least squares [31]. The sum of the squared residuals, *Φ*, was used to compare the accuracy of the fitting results below. The fitting parameters obtained from this calibration exercise were used to calculate parameters for 155°C and 170°C without fitting.

In this model, there exist three unknown rate parameters *k*_*a*_, *k*_*b*_ and *k*_*d*_ (m ^3^ mol ^−1^ s ^−1^) and one unknown material parameter *α*. If *α*=0 then there is no unreactive bagasse, and the standard population balance equation system with no “hard-to-hydrolyse” consideration is resumed. It is noted that there is also some potential uncertainty in the diffusion coefficients which shall be investigated further below. Presently, however, existing formulae and information from the literature are used to estimate the diffusion coefficients in the model.

**Figure 3:**
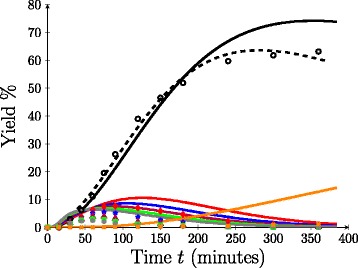
**Yield profiles at 110°C,** ***α*** **=0.** Model yield profiles produced by fitting to only the experimental xylose curve (dashed) and the full set of experimental xylo-oligomer profiles (xylose, oligomers and furfural) (solid). Model results are compared to experimental data (symbol). Key: Xylose (black circle), *i*=2 (red diamond), *i*=3 (blue star), *i*=4 (red violet triangle), *i*=5 (green square), *i*=6 (grey asterisk), furfural (orange multiplication sign).

Firstly, consider the ability of the model to reproduce the experimental xylose profile by fitting only the rate parameters, *k*_*a*_, *k*_*b*_ and *k*_*d*_, assuming *α*=0. The dashed curve in Figure 3 compares the time evolution of the yield (*%*) of xylose monomer as predicted by the model to the experimental yield obtained at 110°C. It is observed that the model and experimental results are well correlated (*Φ*=42.97). Since dilute acid pretreatment is concerned with the removal of solubilised hemicellulose from the bagasse material, such xylose yield curves are commonly used in the literature to describe both the efficacy of the pretreatment and the ability of the model to accurately represent the chemical and physical processes occurring during acid hydrolysis. However, one thing that can influence the accuracy of parameters obtained through fitting is the number of data points used to constrain the parameter space of the model. A model that accurately represents the data when fit to a large number of data points is conceivably more substantiated than the one that fits when only compared to a small number of data points. However, when fitting a model to a single outcome such as xylose yield, even with many data points, we must be careful to only infer that the model accurately portrays how much xylose is produced and not the chemical mechanism behind xylose production.

An important benefit of using population balances in the form of polymer degradation equations is that oligomer yields can be predicted for any chain length. The stringency of the fit is limited by the number of experimentally measurable oligomers in solution, rather than the model itself. Here, an analysis of the ability of the model to compare oligomer yield profiles for chains of length one to six, in addition to the yield of furfural over a range of temperatures, namely 110°C, 125°C and 140°C, is presented. The solid curves in Figure 3 demonstrate the results of using PEST to obtain the best fit between the model and the experimental yields of xylose, oligomers and furfural at 110°C. In fitting the data, *k*_*a*_, *k*_*b*_ and *k*_*d*_ are varied and *α* is set to zero. This approach is therefore the same as that used to fit the model to the xylose yield data without oligomers (dashed curve). However, unlike the model fit to xylose alone, the best fit of the model when all oligomer profiles are used in the fitting criteria does not correlate well (*Φ*=1,182) with any of the experimental profiles. This discrepancy is clear in Figure 3. Therefore, even though the model looked capable of reproducing the xylose curve, it can be seen that under a more stringent fitting regime, the model does not accurately capture the chemical and/or physical processes that are occurring during dilute acid hydrolysis of bagasse fibre, and hence, its usefulness as a predictive tool would seem to be questionable.

**Figure 4:**
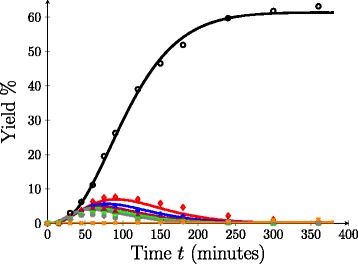
**Oligomer yield profiles at 110°C,** ***α*** **≠0.** Model yield profiles (solid) compared to experimental data (symbol). Key: Xylose (black circle), *i*=2 (red diamond), *i*=3 (blue star), *i*=4 (red violet triangle), *i*=5 (green square), *i*=6 (grey asterisk), furfural (orange multiplication sign).

Figure 3 shows that the model tends to overestimate the experimental yield when fit to oligomer profiles. This is likely because no consideration has been given to the steric and structural obstacles intrinsic to the bagasse material that hinder hemicellulose hydrolysis. The existence of a fast and slow hydrolysing fraction was initially inferred from experimental observations; however, the chemical cause of this phenomena has been speculated upon. Possible sources of hemicellulose recalcitrance include interchain hemicellulose linkages, tethering of hemicellulose to cellulose/lignin, steric hindrance caused by branched chains on the xylan backbone, transport limitations, and to a smaller extent, regions of crystalline hemicellulose [3,24,32,33]. Perhaps, the most accepted hypothesis for the existence of hard-to-hydrolyse kinetics is that portions of the hemicellulose have reduced accessibility, particularly due to interactions with lignin (either though hemicellulose-lignin linkages or hemicellulose embedded within the lignin framework) [30,33]. This is one of the concerns that the “hard-to-hydrolyse” parameter, *α*, is able to address as a simplistic catchall for bagasse hemicellulose recalcitrance. Figure 4 shows the results of using PEST to obtain the best fit between the model and experimental oligomer yield data at 110°C when varying four parameters, *k*_*a*_, *k*_*b*_, *k*_*d*_ and *α*. It is observed that the inclusion of the hard-to-hydrolyse parameter, *α*, results in an improved fit between the model and the experimental data over a broad range of oligomer profiles, where the sum of the squared residuals was reduced to *Φ*=59.01 for non-zero *α* from *Φ*=1182 when *α*=0. The model is able to replicate xylose and furfural profiles to a high degree of accuracy, and it produces significantly better estimates of the longer chain oligomer profiles in comparison to those in Figure 3. It is noted that there is still some inconsistency between the model yield and the experimental yield for the longer oligomer chains, *Y*_4,5,6_. However, this is not wholly unexpected as the effect of any experimental error is magnified when the yields are small.

**Figure 5:**
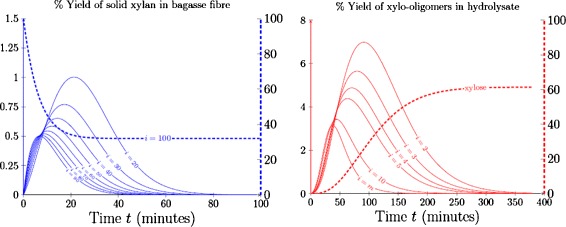
**Sample of possible yield profiles obtained from the population balance framework at 110°C.** Yield of solid chains remaining in the fibre (blue) and yield of aqueous chains obtained in the hydrolysate (red).

Figure 5 provides a sample of the model yield profiles available as a result of using population balances with *α*≠0. The ability to predict solid and aqueous profiles enables theoretical yields modelled by polymer degradation equations to be compared against experimental oligomer profiles obtained from the hydrolysate or experimental determinations of xylan remaining in the solid depending upon the type of data available.

**Figure 6:**
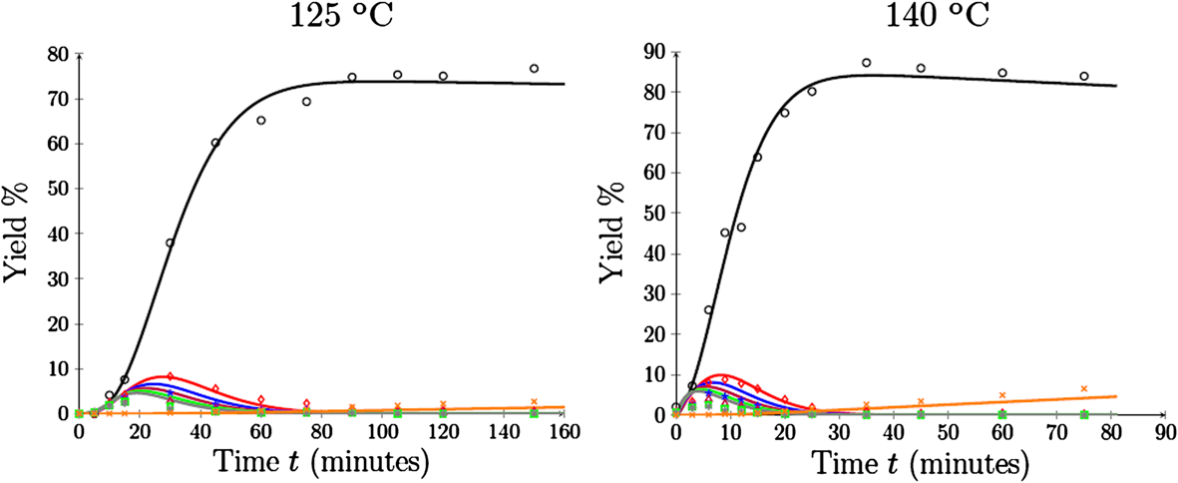
**Oligomer yield profiles for** ***α*** **≠0 at 125°C and 140°C, respectively.** Model yield profiles (solid) compared to experimental data (symbol). Key: Xylose (black circle), *i*=2 (red diamond), *i*=3 (blue star), *i*=4 (red violet triangle), *i*=5 (green square), *i*=6 (grey asterisk), furfural (orange multiplication sign).

The fitting results for *k*_*a*_, *k*_*b*_, *k*_*d*_ and *α* at 125°C and 140°C are shown in Figure 6. The sum of the squared residuals was *Φ*=99.35 at 125°C and *Φ*=318.1 at 140°C. In both cases, the model yield curves were closely aligned with the experimental results for furfural, xylose and xylobiose. Similar to the 110°C results, the model was less accurately able to reproduce the experimental data as the yields became smaller. Despite this, the modelled yields for chains of length three or greater were still able to approximate the time to extinction of each species, which was not the case in Figure 3 when *α* was fixed at zero.

The validity of *α* as a useful fitting tool has been demonstrated; however it is important to determine the value of *α* as a descriptor of the mechanisms of acid hydrolysis. There are two possibilities to consider: firstly, that the introduction of an additional unknown parameter improved the fit simply by increasing the degrees of freedom in the parameter space, or alternatively that *α* improves the fit because it broadly captures some behaviour in the bagasse acid hydrolysis process that is influential to the yield results. To make this distinction, the model was fit with *α* kept fixed (*α*=0), but with the bulk diffusivities, *D*_*∞*_ and $D_{\infty }^{F}$ introduced as additional free parameters, thus increasing the number of free parameters in the model to five. When PEST was used to obtain the best fit between the model and experimental yield data at 110°C where *k*_*a*_, *k*_*b*_, *k*_*d*_, *D*_*∞*_ and $D_{\infty }^{F}$ were allowed to vary, the resultant sum of the squared residuals was *Φ*=1282, similar to that obtained for the case where only three parameters *k*_*a*_, *k*_*b*_ and *k*_*d*_ were fit. Interestingly, it is observed that this permutation of the model is not able to reproduce the experimental results with the same consistency as the “hard-to-hydrolyse” model, Figure 4, despite having an increased degree of freedom in the parameter space. Consequently, these results anecdotally suggest that *α* or some equivalent parameter that characterises the structural properties of hemicellulose in bagasse may be needed when modelling dilute acid pretreatment.

**Figure 7:**
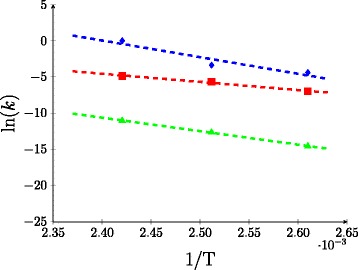
**Arrhenius plot for** ***k***_***a***_ **(blue diamond),** ***k***_***b***_ **(red square) and** ***k***_***d***_ **(green triangle).**

The values of the kinetic parameters and *α* obtained from the data fitting are collated in Table 2, along with the pre-exponetial factors, *k*^0^ (m ^3^ mol ^−1^ s ^−1^) and activation energies, *E*_*a*_ (J mol ^−1^), that were calculated for *k*_*a*_, *k*_*b*_ and *k*_*d*_ from the Arrhenius plot in Figure 7. The temperature dependence of *α*(*T*) was obtained by fitting an exponential curve to the *α* values in Table 2, such that:

(14) $$ {\alpha(T) = 4.1820\times 10^{9}\,\text{exp}\left(-6.0514\times 10^{-2}\,T\right)}  $$

**Table 2:** **Rate parameters**

	***k***_***a***_ ** (m** ^**3**^ ** mol** ^**−1**^ ** s** ^**−1**^ **)**	***k***_***b***_ ** (m** ^**3**^ ** mol** ^**−1**^ ** s** ^**−1**^ **)**	***k***_***d***_ ** (m** ^**3**^ ** mol** ^**−1**^ ** s** ^**−1**^ **)**	***α***
110°C	2.0630 ×10^−4^	1.5434 ×10^−5^	7.9618 ×10^−9^	0.32016
125°C	5.7028 ×10^−4^	5.7128 ×10^−5^	5.3401 ×10^−8^	0.17809
140°C	1.6667 ×10^−2^	1.2914 ×10^−4^	2.6999 ×10^−7^	0.05211
*k* ^0^ (m ^3^ mol ^−1^ s ^−1^)	1.6467 ×10^22^	8.9850 ×10^7^	9.9506 ×10^12^	-
*E*_*a*_ (J mol ^−1^)	1.9129 ×10^5^	9.3422 ×10^4^	1.54675 ×10^5^	-

as demonstrated in Figure 8.

**Figure 8:**
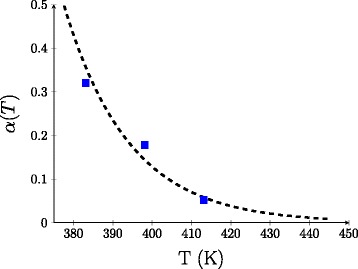
**The “hard-to-hydrolyse” parameter,** ***α*** **(** ***T*** **), over the experimental range of 110°C to 170°C.**

An exponential relationship was chosen in order to ensure that the function approaches zero asymptotically and is thus non-negative at high temperatures. This assumption is in line with work in the literature which suggests that there is no need for separate fast and slow kinetic pathways at high temperatures [30]. It is noted that as temperature decreases, the magnitude of *α*(*T*) increases rapidly (exponentially), and hence, the formulation presented in Equation 14 is not suitable at lower temperatures. Further experimentation is required to determine an expanded temperature profile for *α*(*T*), and without this information, it is difficult to assume the functional form of temperature dependence at temperatures outside the scope of the experimental work conducted here (110°C to 170°C).

**Figure 9:**
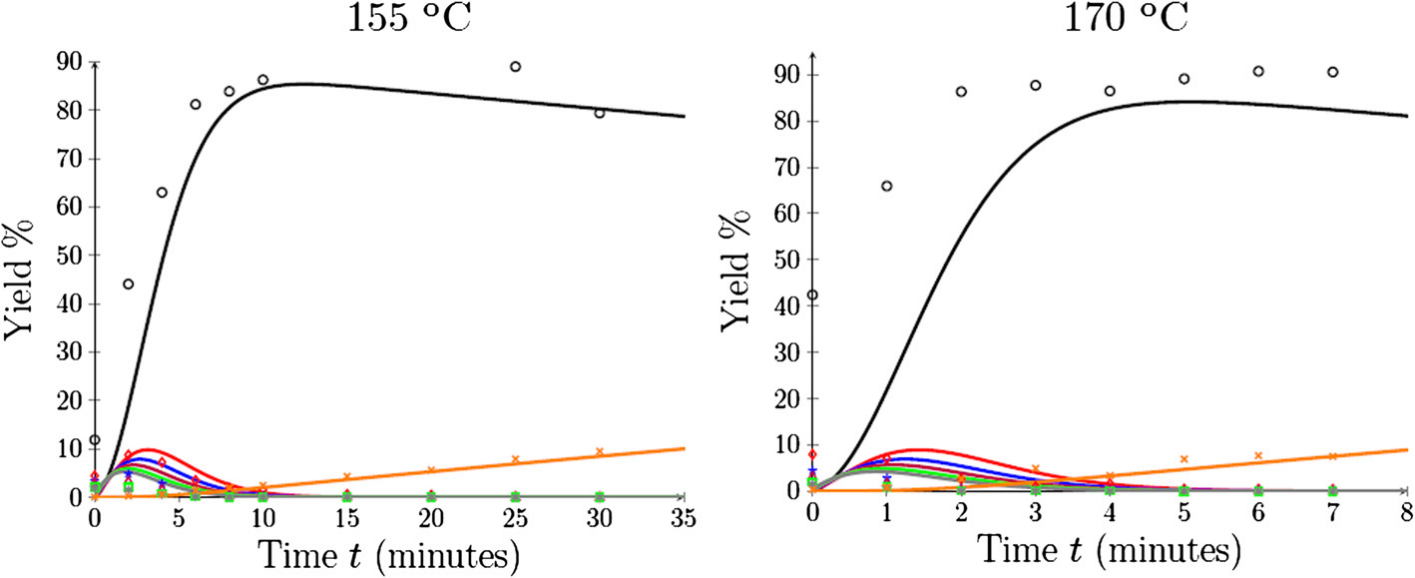
**Predicted xylose, furfural and oligomer yields at 155°C and 170°C, respectively.** Model predicted oligomer profiles (solid) compared to heating compromised experimental data (symbol). Key: Xylose (black circle), *i*=2 (red diamond), *i*=3 (blue star), *i*=4 (red violet triangle), *i*=5 (green square), *i*=6 (grey asterisk), furfural (orange multiplication sign).

As discussed previously, it is difficult to experimentally determine rate parameters in lignocellulosic materials at high temperatures due to the short timescales involved and the limitations of the experimental set-up. Thus, rather than using the yield data measured at 155°C and 170°C to calibrate our model, this information was used to validate the model. To achieve this, the model was executed with the temperature-dependent parameters calculated from Equations 6 and 14 at temperatures of 155°C and 170°C. The resultant model predicted yields were compared to the experimental data recorded at these temperatures, as demonstrated in Figure 9.

It is observed that at both temperatures, the model predictions compare reasonably well to the experimental results. It can be seen that the model predictions appear to be slightly shifted (in time) to the right of the experimental results; however, this was expected given that the long heat up time in the experimental set-up caused the experimental data to reflect that a significant amount of hydrolysis had already occurred by *t*=0.

The results in Figure 9 suggest that the model described in Equations 1 to 5 and 8 to 10, with boundary conditions given by Equation 11 and an initial condition as specified in Equation 12, and with parameters from Equations 6 and 14 is a useful tool for accurately predicting the yield of hemicellulose obtained from the acid pretreatment of sugarcane bagasse.

## Conclusions

A novel mathematical model of the hydrolysis of sugarcane bagasse has been developed in this study that uses population balance kinetics to describe chain degradation, diffusion to account for mass transport of soluble oligomers in solution and conservation of volume arguments to account for the change in porosity of the fibrous material caused by the solubilisation of solid xylan chains. Experimental yield profiles were obtained for the dilute acid hydrolysis of hemicellulose oligomers (*X*_2_– *X*_6_) as well as xylose and furfural. The experimental data obtained was used to calibrate the model by elucidating unknown parameter values through parameter fitting.

Careful consideration must be given to the interpretation of parameters obtained from model fitting when only a single set of data (for example, xylose yield) is used to constrain the fit. The robustness of an acid hydrolysis model can be determined by comparing the model generated yield profiles to a more stringent set of fitting criteria. Such an exercise has been undertaken in this work where the model fit was constrained by oligomer profiles for xylobiose through xylohexaose, in addition to the typical xylose and furfural data sets.

The results showed that adapting “hard-to-hydrolyse” dynamics for a population balance model of acid hydrolysis appears to be able to reproduce yield profiles of not only xylose and furfural but also short-chain oligomers with some degree of accuracy. The model also showed some predictive capability in approximating yield profiles at higher temperatures, where the experimental data was compromised by the heating time of the experimental equipment.

The model presented here has reproduced laboratory scale experimental results. To apply such a model to an industrial “reactor” scale would be largely beneficial in reducing the number of resource intensive hydrolysis experiments required to determine optimal reactor conditions. Further investigation is needed to determine the applicability of this model on such a scale. Although the model appears to capture the chemistry of acid hydrolysis, the industrial scale poses new challenges specific to the reactor design, and some assumptions made at the laboratory scale may need to be revisited. Reactor scale data is needed before any judgements can be made about scaling up this model.

## Methods

### Materials

Sugarcane bagasse was collected from Racecourse Sugar Mill (Mackay Sugar Limited) in Mackay, Australia. Sugarcane bagasse was washed with hot water at 90°C to remove residual sugars to a negligible amount. The washed sugarcane bagasse was air-dried and gently shaken on a sieve having an aperture size of 1.0 cm to remove pith, and the residues were ground by a cutter grinder (Retsch®; SM100, Retsch GmBH, Germany). The milled bagasse was screened, and particles having width range of 0.5 to 1.0 mm were collected and stored for acid hydrolysis. The water mass fraction of the sieved bagasse sample was 6.3 *%*. The mass fractions of glucan, xylan, arabinan, lignin, acetyl and ash in the dry bagasse sample were 43.8 *%*, 20.2 *%*, 3.3 *%*, 27.5 *%*, 2.5 *%* and 2.1 *%*, respectively [34]. Sulphuric acid (98 *%*, mass fraction), xylose (analytical standard) and furfural (99 *%*, mass fraction) were purchased from Sigma-Aldrich (St. Louis, MO, USA). Xylan oligomers standards (xylobiose, xylotriose, xylotetraose, xylopentaose and xylohexaose) were purchased from Megazyme (Bray, Wicklow, Ireland).

### Acid hydrolysis of bagasse samples

Acid hydrolysis of sugarcane bagasse was conducted with a Dionex™ ASE™ 350 Accelerated Solvent Extractor system (Thermo Scientific, Waltham, MA, USA). A glass fibre was placed to the bottom of a 66-mL Dionium™ cell before loading bagasse to the cell. Afterwards, the cell was loosely packed with 5.00 g of milled sugarcane bagasse (4.68-g dry mass). The cell was automatically placed into the oven preheated to the required temperature. Dilute acid (0.5 *%* H_2_SO_4_, mass fraction) was pumped to fill the cell, and the reaction time was counted when the automated cell heat up time had finished. The dilute acid volume pumped into the cell was recorded, which varied slightly between different batches. The temperature used for acid hydrolysis was in a range of 110°C to 170°C in increments of 15°C. After hydrolysis, the cell was purged with nitrogen for 60 s to drain the hydrolysate. The mass of the hydrolysate was recorded. The hydrolysate was stored at −20°C for analysis.

### Determination of xylose oligomer concentrations

High-performance liquid chromatography (HPLC) systems were used to determine concentrations of xylose, xylose oligomers and the xylose degradation product (furfural). One HPLC system (Waters, Milford, MA, USA) equipped with a RPM monosaccharide column (300 × 8.0 mm, Phenomenex, Lane Cove, NSW, Australia), a pump (Waters 1515), a refractive index (RI) detector (Waters 410) and an autosampler (Waters 2707) was used to determine xylose in acid hydrolysed samples. The samples were neutralised with CaCO _3_ prior to HPLC analysis. The temperature for both columns was 85°C and the mobile phase was water, with a flow rate of 0.5 mL min ^−1^. The other HPLC system equipped with an Aminex HPX-87H column (300 × 8.0 mm, Bio-Rad, Richmond, CA, USA), an integrated pump and autosampling system (Waters e2695) and a RI detector (Waters 410) was used to determine xylose degradation product furfural. The samples subjected to determination of furfural were not neutralised. The column temperature was 65°C and the mobile phase was 5 mmol L ^−1^ H _2_*SO*_4_, with a flow rate of 0.6 mL min ^−1^. Xylose oligomers in pretreatment solution were detected by the HPLC system (Waters, Milford, MA, USA) equipped with a Dionex CarboPac™ PA-100 column (BioLCTM 4 × 250 mm, Thermo Scientific, Waltham, MA, USA), an electrochemical detector (Waters 2465) and the pump and autosampling system (Waters e2695, Milford, MA, USA). The mobile phase consisted of solvent A (150 mmol L ^−1^ NaOH) and solvent B (150 mmol L ^−1^ sodium acetate and 150 mmol L ^−1^ NaOH). The column was run at 30°C with a flow rate of 1 mL min ^−1^ using the gradient method according to curve 6 based on the detection waveform from Dionex Technical Note 21 (Thermo Scientific, Waltham, MA, USA). The gradient method started at 86.7 *%* solvent A and 13.3 *%* solvent B (0 to 1 min). The volume ratio of A to B was changed to 0 *%* : 100 *%* over 1 to 30 min, to 86.7 *%* to 13.3 *%* over 30 to 32 min and maintained at this ratio over 32 to 40 min.

### Calculation of furfural, xylose and xylose oligomer yields

The experimental oligomer yields were calculated as a mass fraction. The initial mass (g) of xylan in the bagasse, X _0_, was calculated such that:

(15) $$ {\fontsize{8.3}{12}\begin{aligned} \text{Mass}\, X_{0} = \text{Xylan fraction of bagasse} \times \text{Dry mass of bagasse sample}. \end{aligned}}  $$

The concentration (g L ^−1^) of each oligomer (*X*_*i*_) in the hydrolysate was converted to mass (g) according to the equation:

(16) $$ {\small\begin{aligned} \text{Mass \(X_{i}\)}(t_{n}) &= 1,000 \times \text{Concentration \(X_{i}(t_{n})\)} \times V(t_{n})\\ &\quad \times \frac{\text{{MW}}(X_{i}) - \text{{MW}}(\text{H}_{2}\text{O})}{\text{{MW}}(X_{i})} \end{aligned}}  $$

where *V* is the volume (m ^3^) of the hydrolysate, MW represents molecular weight (g mol ^−1^) and *t*_*n*_ (mins) are the discrete experimental time points. Hydrolysate volume was converted from mass using the density of water at 25°C, 997.047 kg m ^−3^. Consequently, the yield was calculated:

(17) $$ \text{Yield \(\%\)} = 100\times \frac{\text{Mass}\, X_{i}(t_{n})}{\text{Mass}\, X_{0}}.  $$

Similarly, the furfural yield was calculated according to the equation:

(18) $$ {\fontsize{9}{12}\begin{aligned} \text{Yield} \% =100 \times \frac{1,000 \times \text{Concentration \(X_{F}(t_{n})\)} \times V(t_{n}) \times \frac{132}{96}}{\text{Mass}\, X_{0}}. \end{aligned}}  $$

### Numerical methods

The model equations are formed in terms of two continuous variables (space, *r*, and time, *t*) and one discrete variable (chain length, *i*). The equations were non-dimensionalised, and a vertex-centred finite volume scheme was used to discretise the dimensionless spatial variable, reducing the model to a system of ordinary differential equations (ODEs) in dimensionless time [35]. In MATLAB, the SUNDIALS IDA solver was used to implement the discretised differential algebraic equation (DAE) system [36]. The spatial domain consisted of 100 uniformly spaced nodes in the fibre and 250 uniformly spaced nodes in the hydrolysate. The code was vectorised for efficiency in MATLAB, and the banded structure of the Jacobian was utilised to improve runtime and facilitate a real-time implementation of the parameter fitting algorithm. The run time of a single iteration of the code was a few minutes on a desktop PC.

Parameter fitting was completed using the model independent parameter estimation tool (PEST) [31]. The PEST programme was used to identify values of the rate parameters *k*_*a*_, *k*_*b*_, *k*_*d*_ and *α* that produce a fit of the model results to the experimental data at each temperature. For each temperature, the unknown parameters were varied to find the best simultaneous fit of the model yields to a series of oligomer profiles encompassing furfural, xylose and oligomers up to six chain lengths long. The PEST inputs include a template file (.tpl), an input file (.inp), an instruction file (.ins), a parameter value file (.par) and model output file (.out), which were created manually. PEST also requires a model executable file. The observation file (.obf) and the control file (.pst) were created using the INSCHEK and PESTGEN commands, respectively. The control file was edited such that NOPTMAX =30, PHIREDSTP =0.005, NPHISTP =4, NPHINORED =4, RELPARSTP =0.005 and NRELPAR =4 to more closely align with the recommended control data values in the PEST manual. A relative increment of 0.01 was chosen; however, an increment lower bound (DERINCLB) was specified for each parameter due to the small magnitude of the parameters. The parameters themselves were given a zero lower bound to prevent them from becoming negative, and an upper bound to prevent non-physical values. All observation data points were given equal weighting in the least squares calculation. Spline interpolation was used to find model values at the experimental time points, using MATLAB’s inbuilt interp1 function. An Arrhenius plot was used to find the temperature-dependent form of the rate parameters as discussed in the Results section, and excel was used to find the exponential form of *α*(*T*).
